# Trophic niches reflect compositional differences in microbiota among Caribbean sea urchins

**DOI:** 10.7717/peerj.12084

**Published:** 2021-08-31

**Authors:** Ruber Rodríguez-Barreras, Eduardo L. Tosado-Rodríguez, Filipa Godoy-Vitorino

**Affiliations:** 1 Department of Biology, University of Puerto Rico at Bayamón, Bayamón, Puerto Rico, USA; 2 Microbiology and Medical Zoology, School of Medicine, University of Puerto Rico, School of Medicine, San Juan, Puerto Rico, USA

**Keywords:** Sea urchin, Microbiota, Caribbean, Niche

## Abstract

Sea urchins play a critical role in marine ecosystems, as they actively participate in maintaining the balance between coral and algae. We performed the first in-depth survey of the microbiota associated with four free-living populations of Caribbean sea urchins: *Lytechinus variegatus*, *Echinometra lucunter*, *Tripneustes ventricosus*, and *Diadema antillarum*. We compared the influence of the collection site, echinoid species and trophic niche to the composition of the microbiota. This dataset provides a comprehensive overview to date, of the bacterial communities and their ecological relevance associated with sea urchins in their natural environments. A total of sixty-samples, including surrounding reef water and seagrass leaves underwent 16S rRNA gene sequencing (V4 region) and high-quality reads were analyzed with standard bioinformatic approaches. While water and seagrass were dominated by Cyanobacteria such as *Prochlorococcus* and *Rivularia* respectively, echinoid gut samples had dominant Bacteroidetes, Proteobacteria and Fusobacteria. *Propionigenium* was dominant across all species’ guts, revealing a host-associated composition likely responsive to the digestive process of the animals. Beta-diversity analyses showed significant differences in community composition among the three collection sites, animal species, and trophic niches. Alpha diversity was significantly higher among *L. variegatus* samples compared to the other species. *L. variegatus* also displayed an increased abundance of Planctomycetes and Cyanobacterial OTUs. The bacterial community of this herbivorous echinoid reflected similarities to the microfilm community found on *Thalassia testudinum* leaves; a very abundant seagrass and its main food resource. The results of this study elaborate on the microbial ecology of four important Caribbean echinoids, confirming that selection on the microbial community is trophic-niche dependent.

## Introduction

Current knowledge about bacterial communities has grown deeply over the past decades (Fricker, Podlesny & Fricke, 2019). Traditional microbiological methods, such as culture and microscopy, have shown the occurrence of bacteria in gut content microbiota of sea urchins, most of them related with ecological interactions and metabolic processes (Prim & Lawrence, 1975; De Ridder, Jangoux & De Vos, 1985; Temara et al., 1993). Recent advances of molecular sequencing approaches have emerged as powerful tools to revolutionize the characterization of microbiomes in marine animals (Pett, Polage & Schreckenberger, 2005; Nelson et al., 2010; Hentschel et al., 2012; Hakim et al., 2016; Fricker, Podlesny & Fricke, 2019; Rodríguez-Barreras et al., 2020). Current knowledge of the gut microbiome and its benefits have been focused on terrestrial vertebrates, mainly on vertebrates, particularly birds, fish, and mammals, using high-throughput 16S rRNA gene sequencing (Godoy-Vitorino et al., 2012; Gajardo et al., 2016; Hird et al., 2015); but also among marine invertebrates like sponges, mollusk, and cnidarians (King et al., 2012; Krediet et al., 2013; Webster & Thomas, 2016). However, marine invertebrates such as corals, mollusk, arthropods, or echinoderms have received less attention, particularly in the Caribbean (Godoy-Vitorino et al., 2017; Offret et al., 2019; Pagán-Jiménez et al., 2019). Microbiota studies on echinoderms has focus on starfish (Nakagawa et al., 2017), ophiuroids (Dong et al., 2021), sea cucumbers (Pagán-Jiménez et al., 2019; Zhang et al., 2021), and sea urchins (Zhang et al., 2014; Faddetta et al., 2020b; Zuber, 2016; Boraiy, 2018; Ziegler et al., 2020; Faddetta et al., 2020a; Schwob et al., 2020).

Echinoderms are marine invertebrates with more than 7,000 living species and more than 13,000 extinct species distributed in five classes (Hendler et al., 1995). They inhabit different biotopes from the intertidal zone to the abyssal regions in all latitudes, and their presence is relevant in coral reefs and other shallow water ecosystems (Williams et al., 2013). Sea urchins are the most diverse Class within echinoderms; from a total of 108 shallow-water echinoderms listed for Puerto Rico, 19 of them are sea urchins (Rodríguez-Barreras, Sabat & Calzada-Marrero, 2013). Among echinoderms, regular sea urchins are important benthic grazers in marine ecosystems (Guzmán & Cortés, 1993; Bonaviri et al., 2011) and can exert a strong influence in the community structure (Underwood, Kingsford & Andrew, 1991).

The most common echinoids in the Caribbean include *Diadema antillarum* Philippi, 1845, *Lytechinus variegatus* (Lamarck, 1816), *Echinometra lucunter* (Linnaeus, 1758), and *Tripneustes ventricosus* (Lamarck, 1816); two of them, *D. antillarum* and *E. lucunter* are inhabitants of corals and hardground zones (Hendler et al., 1995). The species *L. variegatus* and *T. ventricosus* are usually associated with nearshore seagrass beds dominated by *Thalassia testudinum*, upon which the species graze and ingest macroalgae, animal material, and plant leaves including the associated epibenthic community (Beddingfield & McClintock, 2000; Watts, McClintock & Lawrence, 2013); whereas *D. antillarum* and *E. lucunter* feeds mainly on macroalgae, but also small invertebrates (Lawrence, Lawrence & Watts, 2013).

Research on sea urchins has evolved and highlighted important aspects of their ecology, from taxonomy, reproduction to molecular analysis (Williams et al., 2013). In this study, we present the first microbiota description associated with Caribbean free-living populations of sea urchins by characterizing the gut content microbiota of *D. antillarum*, *L. variegatus*, *E. lucunter* and *T. ventricosus* using a NextGen Illumina MiSeq sequencing technology and bioinformatics tools and associated it with sample location and animal species. To gain insight into differences among microbial communities of four sea urchin species, we sampled at three sites to understand how local conditions could affect microbial assemblages of the four sea urchin species. We hypothesize that (*i*) sites proximity will lead to gut microbial communities similarities among the four sea urchin species, but (*ii*) co-inhabiting species will be more similar in gut content microbiota between them, than among the species of other trophic niches.

## Materials & Methods

### Study site

**Figure 1:**
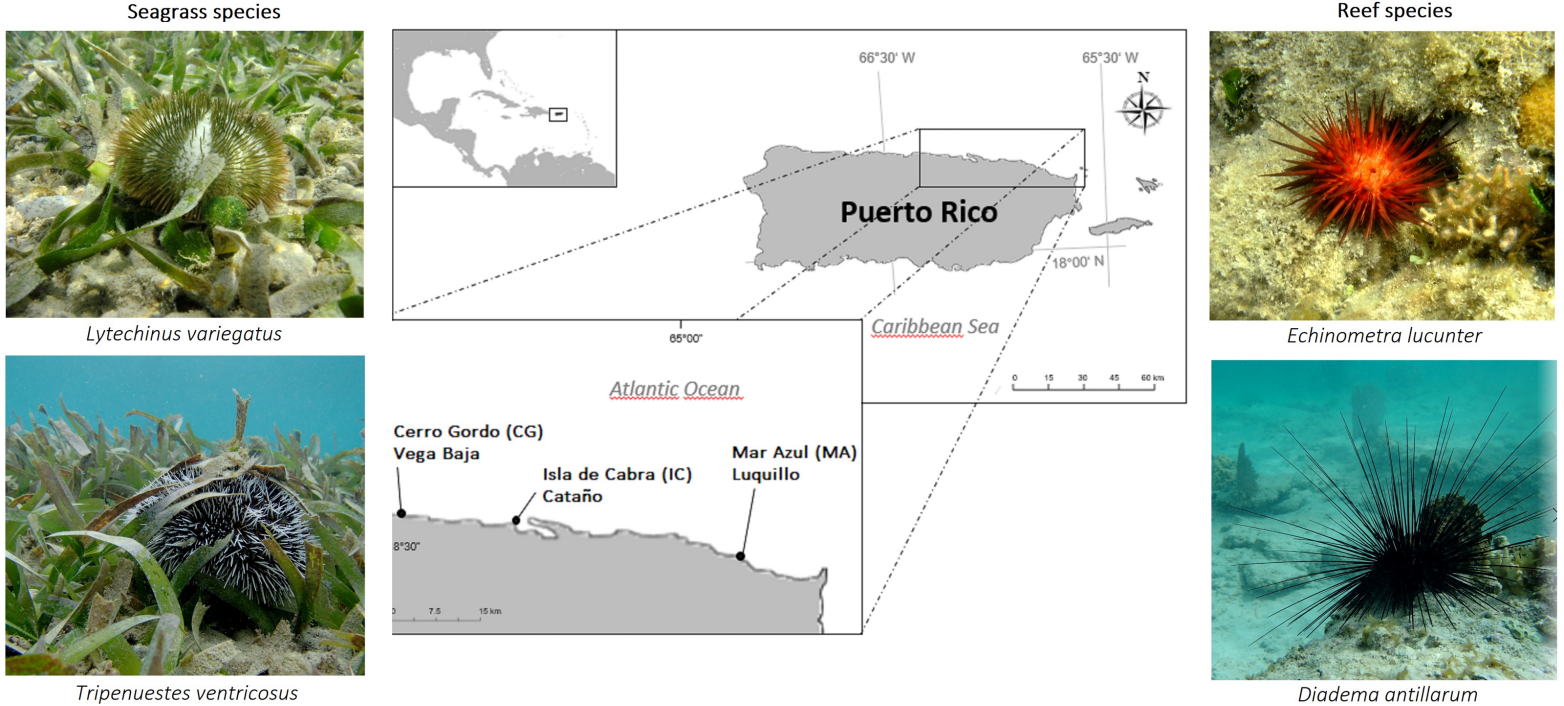
Overview of sample sites in the Northeastern coast of Puerto Rico. Cerro Gordo in Vega Baja (CG), Isla de Cabra in Cataño (IC), and Mar Azul in Luquillo (MA).

The Northern coast of Puerto Rico is characterized by a very narrow shelf and high-energy sandy beaches, due to the action of northeast trade winds and North Atlantic winter storms. Substrate composition of sites are made up of carbonate rocks; and due to the high annual precipitation levels and the discharge of rivers, high sediment discharges are common in the north (Veve & Taggart, 1996). Surveys were conducted during February of 2019 at three shallow-water sites (1-2 m depth) of the Northeastern coast of Puerto Rico (Fig. 1), being the same depth for each species. The sites chosen for the study were Cerro Gordo located in Vega Baja (*CG*, 18°29′05.81″N, −66°20′20.23″W), Isla de Cabra in Cataño (*IC*, 18°28′26.32″N, −66°08′18.82″W), and Mar Azul in Luquillo (*MA*, 18°23′15.01″N, −65°43′11.26″W). Temperature (°C), salinity (‰), and pH measurements were collected *in situ* at each site using the quality meter instrument Pro2030 (https://www.ysi.com/pro2030). We used the average of 5 environmental samples of each parameter per site. Oceanic water conditions were recorded for the three sites, and no differences were found (Table S1).

### Sample collection and processing

Six adults of the species *Diadema antillarum*, *Echinometra lucunter*, and *Tripneustes ventricosus* were collected at all the three sites. Additionally, three adults of *Lytechinus variegatus* were collected in CG and IC, but no individuals of *L. variegatus* were found in MA. The species *E. lucunter* (red urchin) and *D. antillarum* (black urchin) were collected associated to hardground biotopes (fringing reefs), whereas *L. variegatus* (green urchin) and *T. ventricosus* (white urchin) were collected in a back-reef lagoon biotope, covered by seagrass beds of *Thalassia testudinum* leaves, upon which they graze and ingest the leaves. Seawater was collected (1L) in a sterile container from the area where the sea urchins were caught in the reef biotope. Additionally, from the seagrass beds, *T. testudinum* samples were also collected and stored in 50 mL falcon tubes at each site. Sea urchins and seagrass bed samples were put in separate bags with seawater. All samples were put in a foam cooler at the collection site and immediately transported to the lab where they were processed. Sampling of these four species was approved by the Department of Natural and Environmental Resources of Puerto Rico permit number DRNA-2019-IC-003.

In total, 60 animals were anesthetized using 20 mM MgCl_2_, a solution that is used in aquaculture as a suitable nontoxic anesthetic (Arafa, Sadok & Abed, 2007). Animals were first acclimatized in seawater in 100 mL glass beakers with 25 mL seawater for at least 10 min or until they attached as per the approved IACUC protocol A#-5301118. Once attached, an additional 25 mL sterile MgCl_2_ was added. Experimentally induced anesthesia was monitored after a standardized exposure time of 15 min until all sea urchins detached from the walls of the beaker. Once detached, animals were carefully moved by hand to a tray that was placed in a −80 °C ultra-low freezer for 10 mins prior to dissection.

Using flame-sterilized scissors and a metal tray, sea urchins were dissected and opened with an equatorial cut, at the level of the maximum diameter, circumnavigating the mouth. The side along the peristomial membrane was lifted from the sea urchin, while still maintaining the integrity of the digestive tract (Whalen, 2008). The digestive tract (gut tissue, including esophagus, stomach, and intestine, were removed from the sea urchin with scissors, and transferred with a pair of tweezers to a sterilized petri dish. Gut content samples (mostly pellets with few pieces of intestinal tissue) were transferred to 2 ml microtubes and stored in the freezer at −80 °C until DNA extraction procedures. The reef water was filtered using a 0.45 µm membrane device. Membranes then were transferred to a falcon tube and stored at −80 °C for further analyses.

### Genomic DNA extractions

Genomic DNA was extracted from 0.22 µM membranes of filtered seawater, sea grassss and gut fecal pellets from each of the four sea urchin species from reef water and seagrass biotopes (∼200 mg). We used the QIAGEN PowerSoil™ kit (QIAGEN LLC, Germantown Road, Maryland, USA), following manufacturer’s instructions with the following modifications: (1) gut content sample homogenization (3000 r.p.m. for 2 min at room temperature) in a PowerLyzer homogenizer (QIAGEN LLC, Germantown Road, Maryland, USA), and (2) Elution was performed using 100 µl of sterile PCR water previously warmed at 65 °C, to increase DNA yield, allowed to remain on the filter for 5 min incubation at room temperature before the final centrifugation step. A Qubit^®^ dsDNA HS (High Sensitivity) Assay Kit was used to assess DNA concentration (ranging from 5–100 ng/ul) of purified extracts using the Qubit^®^ Fluorometer at room temperature (Waltham, Massachusetts, USA).

### 16S-rRNA gene amplifications and Illumina sequencing

DNA from the gut content samples were normalized to 4 nM during l6S library prep. We amplified the V4 hypervariable region of the 16S ribosomal RNA marker gene (∼291 bp) using the universal bacterial primers: 515F (5′GTGCCAGCMGCCGCGGTAA3′) and 806R (5′GGACTACHVGGGTWTCTAAT3′) in the Earth Microbiome Project (http://www.earthmicrobiome.org/emp-standard-protocols/16s/) (Caporaso et al., 2012) using previously reported conditions (Abarca et al., 2018). We used the Illumina MiSeq Reagent kit 2 ×250 bp to sequence the 16S amplicons. The 16S-rRNA reads were deposited in QIITA (Gonzalez et al., 2018) Bioproject ID 12668, and the raw sequences are available in the European Nucleotide Archive ENA Project: PRJEB40117; ERP123720.

### Bioinformatic analyses and statistical tests

#### Read QC and processing

The 16S rRNA raw FASTQ sequence files were deposited in QIITA (Gonzalez et al., 2018) with its associated metadata information. Raw read pre-processing of demultiplexed files was done with a Phred offset of 33, and default parameters using split libraries FASTQ (QIIMEq2 1.9.1) (Bolyen et al., 2019). Sequences were trimmed to 250 bp and a closed reference approach was selected for OTU picking using the SILVA reference database (Pruesse et al., 2007) for taxonomy assignment with a minimum similarity threshold of 97%. The species table (biom file) was downloaded for downstream analyses using a locally run version of QIIME (Caporaso et al., 2010). Singletons (OTUs with less than three reads), sequences matching chloroplasts, mitochondria, few eukaryotic matches and taxonomically unassigned sequences were removed from downstream analyses.

#### Beta diversity

Community level analyses were done by computing the pairwise Bray-Curtis distances between samples. Global differences in bacterial community composition and structure were visualized with 2D Principal Coordinates Analysis (PCoA) using both sample types, collection sites, niches, and sea urchin species as metadata categories. Additionally, pairwise Bray-Curtis distances between sample sites and sea urchin species were plotted using non-metric multidimensional scaling (NMDS). Statistical significance between sample groups was assessed using the PERMANOVA test (Anderson, 2001). Additionally, ANOSIM, a non-parametric statistical test was used to compare ranked beta diversity distances between different group depths found in the mapping file and calculates a *p-value* based on the Bray-Curtis table used to generate the plots. These tests were done using the script compare_categories.py for each specific test in QIIME (Kuczynski et al., 2011) with the distance matrix as the input file and 999 permutations.

### Alpha diversity and taxonomic plots

Alpha diversity measures of Chao 1 (richness), were plotted as rarefaction curves and boxplots. For alpha diversity statistical tests, we used the script compare_alpha_diversity.py in QIIME, to compare the diversity between groups of samples in each metadata category. These statistical tests were nonparametric t-tests with Monte Carlo permutations to determine the *p*-value. We considered a rarefaction level of 6,700 reads for all the 60 samples together including water and seagrass, while for analyses of gut samples (*n* = 54) the rarefaction level was 7,000 reads. Barplots revealing phyla were computed using QIIME (Caporaso et al., 2010) and those at the genus-level with MicrobiomeAnalyst (https://www.microbiomeanalyst.ca/) using the same parameters.

Additionally, we used the group_significance.py script in QIIME, which compares OTU frequencies across animal species, to ascertain whether or not there are statistically significant differences between the OTU abundance in the different groups (using a Kruskal-Wallis test) S elected boxplots of significantly different taxa , were generated using ggplot2 (Wickham, 2009) (Team, 2020) and taxa with *p*-values <0.05 were marked with an asterisk. The core microbiome was calculated in QIIME and accounted for those OTUs shared by 50% of the samples across the four species. The list of OTUs was added to a web-based tool for Venn diagrams and plotted using http://www.interactivenn.net/index.html (Heberle et al., 2015).

All statistical test results are summarized in (Table S2).

## Results

A total of 4,317,304 of 16S rRNA raw reads were obtained. Removal of sequences due to quality assessment and trimming, removal of singletons, chloroplasts and mitochondria resulted in 2,783,666 good-quality sequence reads. These reads produced 38,117 ± 529.29 OTUs from all 60 samples, including seagrass and water (Table 1), and 29,060 OTUs from the 54 animal gut content samples at three sites in the Northeastern coast of Puerto Rico (Table 2). Isla de Cabra (IC) exhibited the highest number of reads and OTUs, while Cerro Gordo (CG) was the site with less reads and OTUs among the three sites (Table 2). Pre-rarefaction, among the four sea urchin species, *E. lucunter* reached the highest number of reads and OTUs with 972,904 ± 30,979.07 and 9,890 ± 239.74 respectively, followed by *D. antillarum*, *T. ventricosus* and, lastly *L. variegatus* with only 155,592 ± 45,228.63 reads and 2,562 ± 527.10 OTUs (Table 2, Table S2).

### Echinoid-associated microbiota is distinct from environmental samples

**Figure 2:**
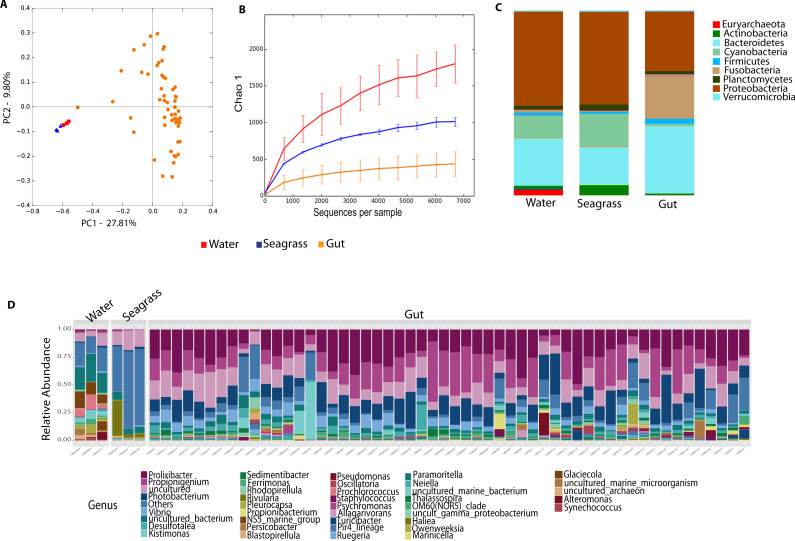
Diversity analyses comparing sample types, including water, seagrass and gut samples from the four sea urchin species. Bray-Curtis analysis represented by a 2D Principal Coordinates Analysis (PCoA) using sample types as metadata categories (A). PERMANOVA showed significant differences in beta diversity (PERMANOVA *p*-value = 0.01, ANOSIM *p*-value =0.001). T-tests on the alpha diversity curves demonstrated that seawater and seagrass environments were significantly more diverse than gut samples (*p*-value = 0.003). Bar Plots depict relative abundance of bacteria at the phyla (C), and genus levels (D).

Environmental (water and seagrass samples) separate clearly from host-associated sea urchin digesta (permanova *p*-value = 0.001; ANOSIM, *p*-value = 0.01; Fig. 2A, Table S2). Alpha diversity was significantly different between seagrass and echinoid samples (*t*-test, *p*-value = 0.003), similarly to reef water and gut samples (*t*-test, *p*-value = 0.003, Fig. 2B), but no remarkable differences were found between reef water and seagrass samples (*t*-test, *p*-value = 0.336). Composition was similar between reef water and seagrass in terms of the relative abundance of Proteobacteria and Bacteroidetes; while Euryarchaeota was only found in water samples and Cyanobacteria was more abundant in seagrass samples. Seagrass samples were dominated by the cyanobacteria *Rivularia*, while in contrast water samples were dominated by various groups such as Rhodopirellula, Paramoritella, NS5 marine group or Prochlorococcus (Fig. 2C). Gut samples had dominant *Prolixibacter*, *Propionigenium*, *Photobacterium* and *Desulfotalea*; while water samples were dominated by *Paramoritella*, *Blastopirellula*, *Prochlorococcus*, NS5 marine group, and other uncultured bacteria, while seagrass samples were dominated by *Rivularia* (Fig. 2D).

**Table 1:** Total number and mean of sequences and Operational Taxonomic Units by species collected at three sites in the northeastern coast of Puerto Rico.

**Sample type**	**# samples**	**SUM of reads**	**Mean ± S.D of reads**	**SUM of OTUs**	**Mean ± SD of OTUs**
*Diadema antillarum*	16	580613	36,288 ± 25,306.50	8505	532 ± 241.89
*Echinometra lucunter*	18	972904	54,050 ± 30,979.07	9890	549 ± 239.74
*Tripneustes ventricosus*	17	739215	43,483 ± 47,473.66	7108	418 ± 202.20
*Lytechinus variegatus*	3	155592	11,665 ± 45,228.63	2562	2,562 ± 527.10
water samples	3	300346	100,115 ± 85,955.07	7564	2,521 ± 834.07
seagrass samples	3	34996	11,665 ± 4,489.03	2488	829 ± 138.12
Grand Total	60	2783666	46,394 ± 40,356.13	38117	635 ± 529.29

**Table 2:** Number of samples, reads and OTUs of the gut microbiota of echinoids according to sample site and species.

**Sample site / Collected species**	**Number of samples**	**Number of reads**	**Sum of OTUS**
**Cataño**			
*Diadema antillarum*	5	214,881	2,503
*Echinometra lucunter*	6	397,791	4,135
*Lytechinus variegatus*	3	155,592	2,562
*Tripneustes ventricosus*	6	463,064	2,993
Total	20	1,231,328	12,193
**Cerro Gordo**			
*Diadema antillarum*	5	235,322	3,435
*Echinometra lucunter*	6	236,659	3,286
*Tripneustes ventricosus*	6	70,961	2,288
Total	18	542,942	9,009
**Luquillo**			
*Diadema antillarum*	6	221,377	3,562
*Echinometra lucunter*	6	338,454	3,562
*Tripneustes ventricosus*	5	205,190	1,827
Total	17	765,021	7,858
**Grand Total**	54	2,539,291	29,060

### Echinoid collection sites and species explain differences in the microbiota *L. variegatus,* an herbivorous echinoid, has the most distinct microbiota

**Figure 3:**
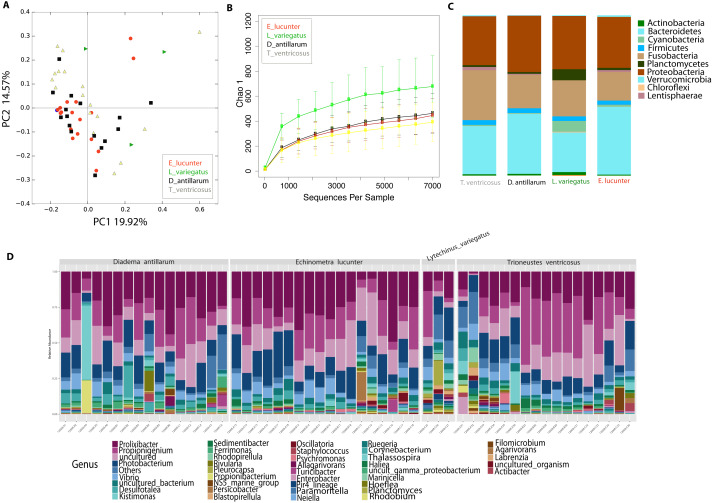
Diversity analyses comparing the four sea urchin species. Bray-Curtis analysis represented in a 2D Principal Coordinates Analysis (PCoA) using species as metadata categories, depicts distinct species clustering with PERMANOVA *p*-value =0.004; ANOSIM *p*-value =0.01 (A). Rarefaction curves of Chao1 index demonstrated significant differences between green (Lytechinus_variegatus) and red (Echinometra_lucunter) sea urchin (*p*-value =0.006) and between red (Echinometra_lucunter) and black (Diadema_antillarum) sea urchin (*p*-value =0.048) (B). Species relative abundance at phyla (C) and genus levels (D) are depicted by the bar plots.

Differences between community structure were detected among sea urchin species (PERMANOVA *p*-value = 0.004; ANOSIM, *p*-value = 0.048). Alpha diversity was significantly higher among *L. variegatus* compared to *E. lucunter* (*t*-test, *p*-value = 0.006) and between *L. variegatus* and *D. antillarum* and (*t*-test, *p*-value = 0.012; Fig. 3B, Table S2). The relative abundance of the microbiota at the phyla-level showed higher dominance of Planctomycetes, Actinobacteria and Cyanobacteria in *L. variegatus* compared to the other species. There were similar amounts of Bacteroidetes, Proteobacteria and Fusobacteria among all species (Fig. 3C). The most abundant bacterial genera were *Prolixibacter*, *Propionigenium* and *Photobacterium*, but in some samples of *D. antillarum* and *T. ventricosus*, *Kistimonas* were particularly abundant (Fig. 3D).

**Figure 4:**
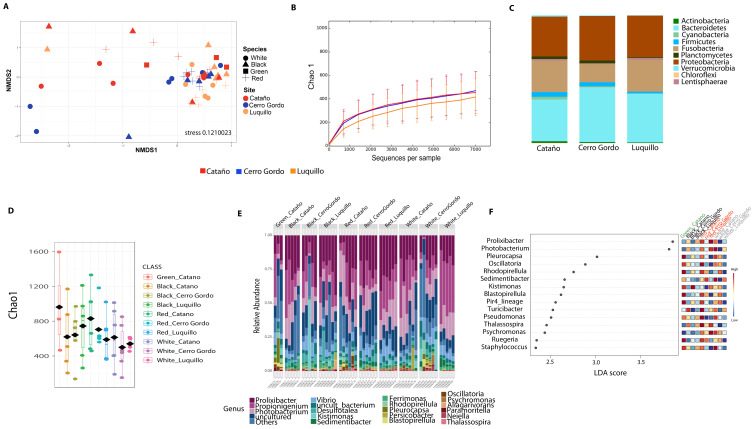
Diversity analyses comparing the three sampling sites: Cerro Gordo, Cataño and Luquillo. Bray-Curtis analysis, represented by an NMDS (stress 0.121003), used collection sites and sea urchin species as metadata categories. PERMANOVA showed significant differences in beta diversity (*p*-value = 0.001, ANOSIM *p*-value = 0.01) (A). Alpha diversity as shown by the rarefaction curves did not demonstrate any significant differences between sites (B). Bar Plots depict relative abundance at phyla level (C). When combining species and site, richness was not significantly different although the green sea urchin (*Lytechinus_variegatus*) seems more diverse (D). Bacterial genus-level plots among species distributed by site are depicted in (E). LEFSE shows genus level taxa that are more dominant per each species distributed by collection site (F).

Community analyses by animal collection site (location), showed significant differences among the three sites, PERMANOVA *p*-value =0.001; ANOSIM, *p*-value = 0.01). Microbiota from animals collected in Luquillo clustered together in axis 1, while axis 2 separated mostly Cerro Gordo from Cataño (Fig. 4A, Table S2). Alpha diversity analyses showed less richness in Luquillo samples, nevertheless, there were no significant differences among sites (Fig. 4B, Table S2). At the Phyla level within all sites, dominant groups included Bacteroidetes, Fusobacteria, and Proteobacteria. Fusobacteria were reduced in samples collected in Cerro Gordo. Bacteroidetes increased in Cerro Gordo, which separated in the NMDS from the other sites (Figs. 4A, 4C). The Phyla Cyanobacteria and Bacteroidetes were more abundant in Cataño (where *L. variegatus* was collected). All species collected from Cataño or Cerro Gordo presented higher richness on their samples indifferently of the sea urchin species Fig. 4D). At the genus level, we found that *Prolixibacter* (Bacteroidetes), *Propionigenium* (Fusobacterium), *Photobacterium* and *Vibrio* (Proteobacteria) were present across all samples (Fig. 2E). The genus *Prolixibacter* was more abundant in *Echinometra lucunter* samples from Cataño and Luquillo (Fig. 4F), and *Photobacterium* were more abundant in IC and MA (Fig. 4F). Only 161 OTUs were considered core among all four echinoids, when computing the core microbiota of OTUs present in 50% of the samples Fig. S1).

### Reef species *D. antillarum* (black echinoid) and *E. lucunter* (red echinoid) have a distinct microbiome from *L. variegatus* (green echinoid) and *T. ventricosus* (white echinoid) occupying the seagrass niche

**Figure 5:**
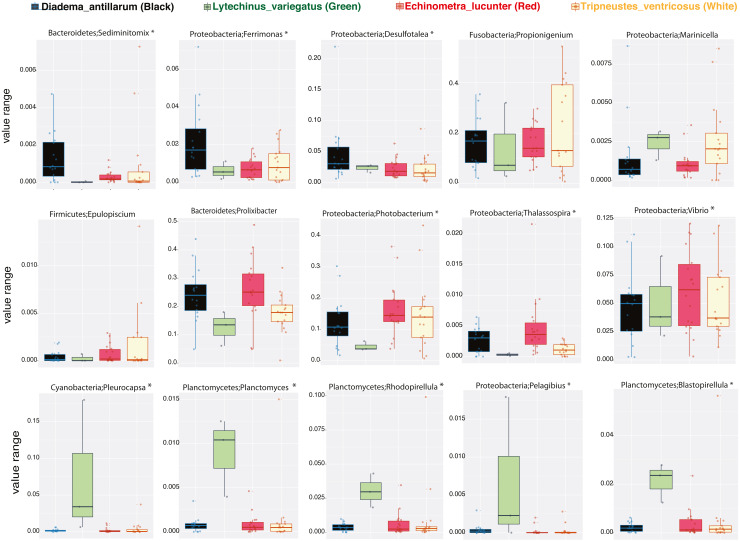
Boxplots of bacterial genus-level analyses (assigned per phylum) that discriminate among the four sea urchin species (*p*-values < 0.05 marked with an asterisk).

**Figure 6:**
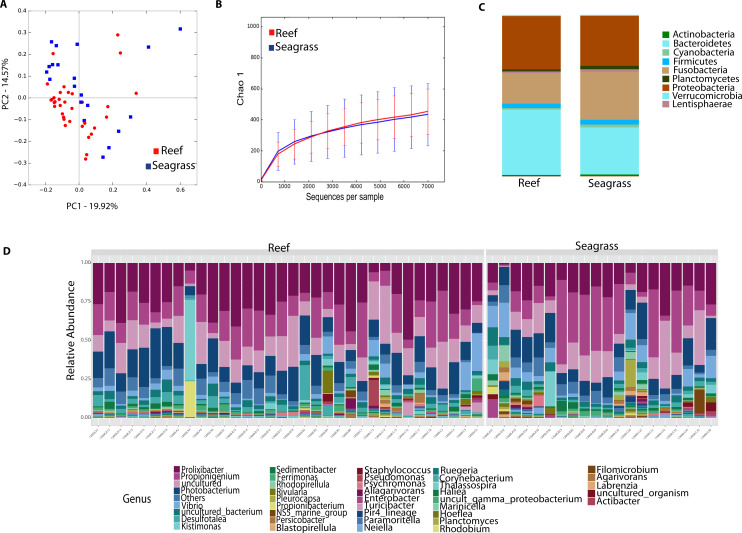
Diversity analyses comparing sea urchin trophic niches (animals collected in the reef or among seagrass). Bray-Curtis analysis is represented by a 2D Principal Coordinates Analysis (PCoA) PERMANOVA *p*-value = 0.007; ANOSIM *p*-value = 0.01 (A). Chao 1 alpha diversity rarefaction curves show no significant differences between trophic niches (*p*-value = 0.704) (B). Bar Plots depict relative abundance at phyla (C), and genus-levels (D).

We then determined the taxonomic biomarkers significantly associated (*p* < 0.05) with each of the four sea urchin species. *D. antillarum* had *Sedimitomix*, *Ferrimonas*, and *Desulfotalea.* Another species collected in the reef niche was *E. lucunter,* dominated by *Thalassospira* and *Vibrio* and shared dominant *Prolixibacter* and *Photobacterium* with *D. antillarum* (Fig. 5). The species *T. ventricosus* exhibited higher relative abundance of *Propionigenium* with respect to the other three sea urchins, but not significantly. *L. variegatus, also* collected in the seagrass bed, had a significantly higher dominance of *Pleurocapsa*, *Planctomyces*, *Rhodopirellula*, *Pelagibius*, and *Blastopirellula* (Fig. 5) and the highest number of unique OTUs, when considering a core microbiome of 50% (Fig. S1). Interestingly, its core microbiota shared a similar number of OTUs with *D. antillarum* and *E. lucunter* (Fig. S1). Certain similarities in species composition among the species collected in the different niches (seagrass and reef) determined significant differences in beta diversity (PERMANOVA *p*-value = 0.007; ANOSIM, *p*-value = 0.01; Fig. 6A, Table S2) but no differences in alpha diversity (Fig. 6B). It comes with low R values associated with ANOSIM, confirming the low dissimilarity (some overlap) between the groups of samples. Samples from seagrass biotopes displayed higher abundances of the phyla *Fusobacteria*, *Lentisphaerae*, and *Planctomycetes*, while having lower concentrations of *Verrucomicrobia*. In contrast, *Bacteroidetes* was more abundant in sea urchin species from reef niche (Fig. 6C). The most abundant genera were *Prolixibacter* in the reef niche, *Propionigenium* and *Photobacterium* at both trophic niches, but mostly in the seagrass bed (Fig. 6D).

## Discussion

This study represents the first known report characterizing the bacterial community of four Caribbean echinoids collected from their natural habitats. Despite our three sampling sites being located in the northeastern zone of Puerto Rico, and physico-chemical parameters (temperature, pH, and salinity) were similar among them, we still were able to detect differences in the gut microbiota per species in each site. These differences found mostly in Luquillo, in comparison to Cataño or Cerro Gordo could be associated with the position where the individuals were collected. All three species collected in Luquillo were closer to each other than the animals collected in the other three sites. For example, individuals of *T. ventricosus* (seagrass biotope) were grazing less than 5 m from the border between the seagrass beds and the back reef zone, which is very close to where *D. antillarum* and *E. lucunter* individuals inhabit. This proximity seems to be key to understanding the similarities among gut microbial communities in Luquillo, with respect to a wider gut microbial distribution found in Cataño and Cerro Gordo. In addition, samples collected in Cerro Gordo, who also separate in NMDS, may derive its differences due to the occurrence of a mangrove lagoon with a canal at 270 m for the collecting site with a nutrient input in nearshore waters (Zieman, Macko & Mills, 1984; Rezende et al., 1990), that could lead to changes in local coastal microbial communities arrangements.

Despite the location and its physical surroundings, factors such as feeding strategies or migration patterns could contribute to the composition of the microbiota. Despite the generalist feeding behavior displayed by sea urchins—where individuals graze the surface and incorporate the crushed material, the co-occurrence of three of the four species in the sampling site—could lead to similarities in microbiota. The core microbiome considering OTUs present in 50% of the samples demonstrated a high number of shared taxa and evidenced common bacteria in individuals sharing the same trophic niche, with emphasis to those collected in the reef, while also highlighting the uniqueness of *L. variegatus*. Hardgrounds and reefs dominated by cnidarians are usually the common habitat of *D. antillarum* and *E. luncunter*. Both co-inhabiting species ingest macroalgal material and small invertebrates while grazing; this generalist feeding behavior is common in sea urchins (Lawrence, Lawrence & Watts, 2013) and may underlie their similar gut microbiomes. The lack of feeding selectivity may increase gut content microbiota similarities between co-inhabiting species as we found in this study, mainly with *D. antillarum* and *E. lucunter*. Hence, a high organic matter flux associated with samples closer to sediments, is usually associated with a depletion in O_2_, thus a dominance in sulfate reducers (*Desulfotalea*) (Thamdrup, Fossing & Jørgensen, 1994) and selenate-reducing bacteria (*Ferrimonas)* (Tertschnig, 1989) as well as other sediment bacteria (Leloup et al., 2009; Carr et al., 2015; Wasmund, Mußmann & Loy, 2017; Anantharaman et al., 2018) were found dominant in the gut of these reef species, contributing to ecological recruitment of marine microbial colonization (Ramírez et al., 2016).

Seagrass biotopes are mainly present in coastal marine waters (Mtwana Nordlund et al., 2016), as those found around Puerto Rico. The species *L. variegatus* and *T. ventricosus* share the same biotope and certain core microbes, nonetheless, the overall microbiota of *T. ventricosus* was more similar in structure and at the phyla-level with *D. antillarum* and *E. lucunter*, both inhabiting the hardground biotopes dominated by coral and other cnidarians. Cyanobacteria were only found in *L. variegatus* as well as a dominance in Planctomycetes. Cyanobacteria likely come from the ingestion of seagrass compared to *T. ventricosus* (Tertschnig, 1989; Hendler et al., 1995), and Planctomycetes are dominant components of the epibiotic marine algae microbiota (Bondoso et al., 2017). This adds to our recent dietary survey which confirmed higher herbivory for *L. variegatus* (Rodríguez-Barreras et al., 2020). *T. ventricosus* usually grazes on *T. testudinum* leaves (Keller, 1983; Barrios & Reyes, 2009), however the species also migrates to the backreef zone where *D. antillarum* and *E. lucunter* inhabit (Moses & Bonem, 2001). This migration pattern across the reef might be causing the differences observed in gut microbial communities between *L. variegatus* and *T. ventricosus*, and similarities with *T. ventricosus*, *E. lucunter* and *D. antillarum*. In fact, most of the collected *T. ventricosus* were found in the border zone between seagrass and backreef biotope, mainly in Luquillo, which may explain our results.

The sea urchin *L. variegatu* s has been targeted in other studies (Hakim et al., 2015; Hakim et al., 2016; Hakim et al., 2019), from individuals kept in aquariums or cultured *versus* wild individuals collected in situ. This is the first study on these species in the Caribbean region, adding to biodiversity surveys of wild echinoids. An analysis of the gut microbiota in *L. variegatus* under a controlled environment, reported a high abundance of *Epsiloproteobacteria* within the order Campylobacterales (Hakim et al., 2015); whereas our study revealed a higher abundance of Bacteroidetes, Fusobacteria, and Proteobacteria in wild samples of *L. variegatus.* Therefore, these discrepancies in composition are due to differences between wild and cultured individuals, which suggest that sample source is a strong influence on the gut microbial community structure (Hakim et al., 2015). Similar to our results, another recent study in Florida, showed that wild *L. variegatus* exhibited higher abundances of Proteobacteria (Gammaproteobacteria) and Bacteroidetes (Hakim et al., 2016). Changes of the gut microbiota between echinoids from the same species between Florida and Puerto Rico demonstrate the geographical impact in the composition and structure of the gut microbial communities.

Overall, phyla level composition was with dominant Bacteroidetes and Proteobacteria, is also present in other echinoderms: *Echinus esculentus* (Unkles, 1977), *Strongylocentrotus droebachiensis* and *Tripneustes ventricosus* (Guerinot & Patriquin, 1981) as well as sea cucumbers (Pagán-Jiménez et al., 2019). Additionally, our in-depth sequencing detected other phyla including Fusobacteria, Cyanobacteria and Planctomycetes, the latter mostly in the herbivorous *L. variegatus* as well as significant genus-level differences among species and sites. Cyanobacteria such as *Rivularia* and *Prochlorococcus* are primary producers in the ocean (Chisholm et al., 1992). Gut samples from all sea urchin species were dominated by *Prolixibacter*, *Propionigenium*, and *Photobacterium*, previously described in other sea urchin species (Yao et al., 2019). *Prolixibacter* are facultative anaerobes (Holmes et al., 2007) while *Propionigenium* are obligate anaerobes and key players in the metabolism succinate and propionate (Schink, 2006), likely being important players in the digestion process of these Caribbean echinoids.

## Conclusions

This is the first high-throughput study characterizing the gut microbial community composition in four, wild caught, Caribbean sea urchin species using NextGen sequencing. Collection site and phylogenetic species explained the differences among the microbiota, with certain commonalities among those sharing a trophic niche. Reef-associated urchins displayed more abundance of sulfate reducing bacteria while those inhabiting seagrass beds had dominant Planctomycetes and Cyanobacteria, revealing its herbivorous diet. All individuals presented dominant *Propionigenium,* fermentative bacteria likely involved in the digestion of these animals. Future studies must focus on the characterization of the microbiota of other parts of the digestive system, such as the pharynx and esophagus, as well as on the composition of the epidermal and the coelo-microbiota. A compartmentalized approach would allow us to determine what groups of bacteria play a role in the digestive process by gut section and coupled with shotgun metagenomics, could reveal the extent of genes and metabolic pathways of the microbiota in the gut of these echinoids.

##  Supplemental Information

10.7717/peerj.12084/supp-1 Supplemental Information 1 Local oceanic water conditions at three localities in the northeastern coast of Puerto Rico

10.7717/peerj.12084/supp-2 Supplemental Information 2 Non-parametric *t*-test comparing observed richness between the different metadata categories

10.7717/peerj.12084/supp-3 Supplemental Information 3 Venn diagram of shared core OTUs among the four echinoid species We calculated the core 50% (OTUs present in 50% of the samples) for each species.

## Additional Information and Declarations

### Field Study Permissions

The following information was supplied relating to field study approvals (i.e., approving body and any reference numbers): Department of Natural and Environmental Resources of Puerto Rico approved this research (DRNA-2019-IC-003).

### DNA Deposition

The following information was supplied regarding the deposition of DNA sequences: The 16S-rRNA reads are available in the QIITA Bioproject ID 12668, and the raw sequences are available in the European Nucleotide Archive: PRJEB40117; ERP123720.

### Data Availability

The following information was supplied regarding data availability: The 16S-rRNA reads are available in QIITA BioProject ID 12668: https://qiita.ucsd.edu/study/description/12668.
