# Supplementary material for: Trophic niches reflect compositional differences in microbiota among Caribbean sea urchins
Source: PeerJ. 2021 Aug 31;9:e12084. doi: 10.7717/peerj.12084 (PMC8415288; doi:10.7717/peerj.12084)
Supplement: Supplemental Information 1 [file peerj-09-12084-s001.docx]

**Supplementary Table 1.** Local oceanic water conditions at three localities in the northeastern coast of Puerto Rico.

| **Sites** | **Temp (**ºC**)** | **Salinity (**o/oo**)** | **pH** |
| --- | --- | --- | --- |
| CG | 25.6 ± 0.00 | 33.32 ± 0.04 | 8.45 ± 0.05 |
| IC | 26.08 ± 0.08 | 33.56 ± 0.05 | 8.41 ± 0.08 |
| MA | 26.76 ± 0.07 | 33.8 ± 0.16 | 8.32 ± 0.05 |
