# Supplementary figures and images for: Trophic niches reflect compositional differences in microbiota among Caribbean sea urchins
Source: PeerJ. 2021 Aug 31;9:e12084. doi: 10.7717/peerj.12084 (PMC8415288; doi:10.7717/peerj.12084)

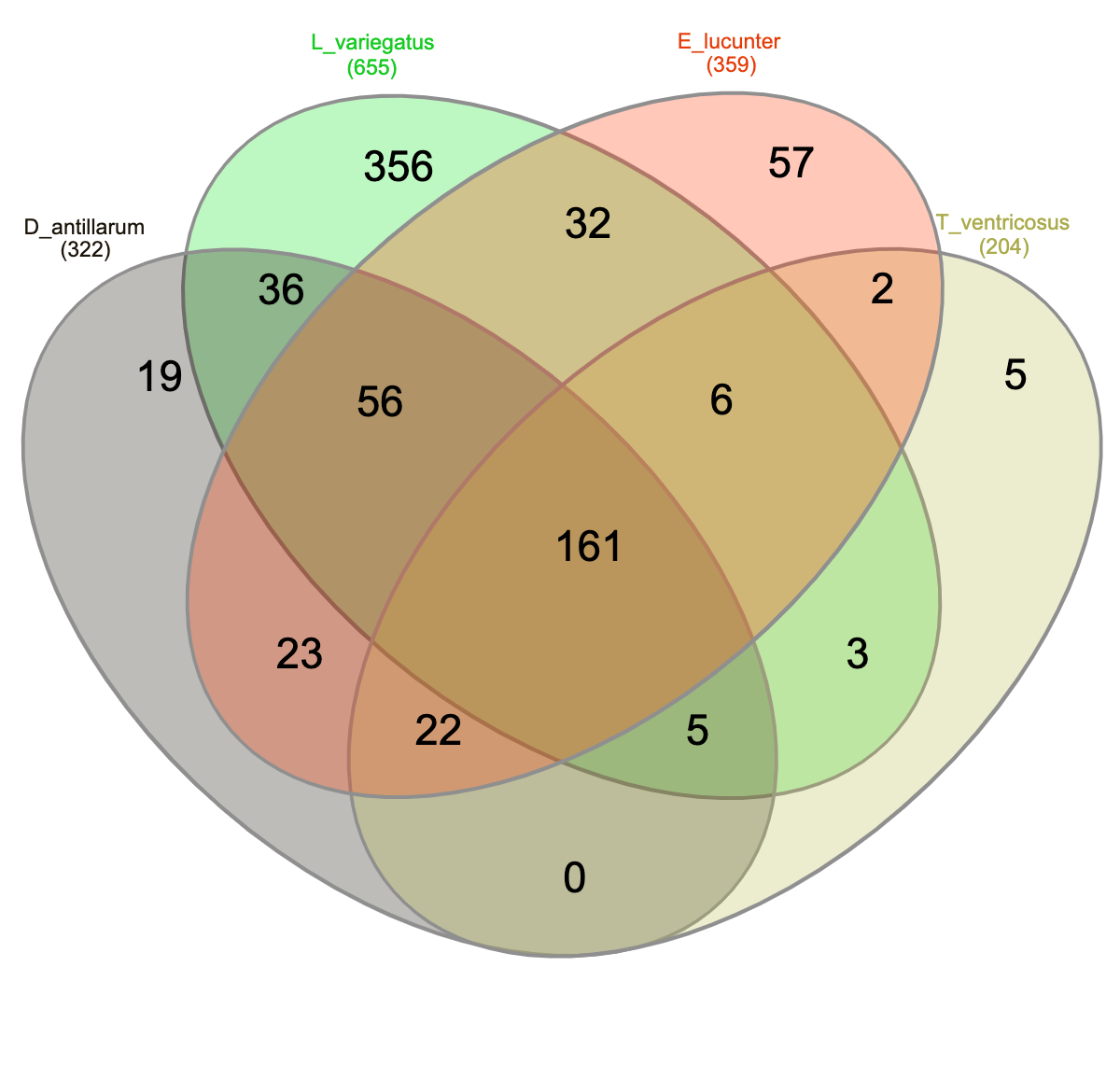

Supplement: Supplemental Information 3 — We calculated the core 50% (OTUs present in 50% of the samples) for each species. [file peerj-09-12084-s003.png]
